# Supplementary material for: Mitochondrial dynamics and segregation during the asymmetric division of Arabidopsis zygotes
Source: Quant Plant Biol. 2020 Nov 30;1:e3. doi: 10.1017/qpb.2020.4 (PMC10095797; doi:10.1017/qpb.2020.4)
Supplement: Supplementary file 1 [file qpbsup.zip › S2632882820000041sup004.docx]

**Supplementary Materials**

Supplementary Movies S1 to S3

**Supplementary Movie S1. Mitochondrial dynamics from the young zygote to the first cell division.**

Time-lapse observation of the mitochondrial/nuclear marker in the zygote. Numbers indicate the time (h:min) from when the zygote started elongation. MIP images are shown, and images were obtained at 20-min intervals.

Scale bar: 10 µm.

**Supplementary Movie S2. Mitochondrial dynamics during zygote cell division.**

Time-lapse observation of the mitochondrial/nuclear marker from the mature zygote to the zygote division. Numbers indicate the time (h:min) from the observation began. MIP images are shown, and images were obtained at 10-min intervals.

Scale bar: 10 µm.

**Supplementary Movie S3. The effect of oryzalin on the mitochondrial dynamics during zygote cell division.**

Time-lapse observation of the mitochondrial/nuclear marker in the zygote after the addition of oryzalin to the *in vitro* ovule cultivation media. Numbers indicate the time (h:min) from the observation began. MIP images are shown, and images were obtained at 20-min intervals.

Scale bar: 10 µm.
